# Synergistic combination of doxorubicin with hydralazine, and disulfiram against MCF-7 breast cancer cell line

**DOI:** 10.1371/journal.pone.0291981

**Published:** 2023-09-28

**Authors:** Zainab Lafi, Walhan Alshaer, Lobna Gharaibeh, Dana A. Alqudah, Baidaa AlQuaissi, Banan Bashaireh, Abed Alqader Ibrahim

**Affiliations:** 1 Pharmacological and Diagnostic Research Centre, Faculty of Pharmacy, Al-Ahliyya Amman University, Amman, Jordan; 2 Cell Therapy Center, The University of Jordan, Amman, Jordan; 3 Department of Nanoscience, Joint School of Nanoscience and Nanoengineering, University of North Carolina at Greensboro, Greensboro, NC, United States of America; IHRC, Inc. (Human Resource Service Administration), UNITED STATES

## Abstract

Disulfiram and hydralazine have recently been reported to have anti-cancer action, and repositioned to be used as adjuvant in cancer therapy. Chemotherapy combined with other medications, such as those that affect the immune system or epigenetic cell profile, can overcome resistance with fewer adverse effects compared to chemotherapy alone. In the present study, a combination of doxorubicin (DOX) with hydrazine (Hyd) and disulfiram (Dis), as a triple treatment, was evaluated against wild-type and DOX-resistant MCF-7 breast cancer cell line. Both wild-type MCF-7 cell line (MCF-7_WT) and DOX-resistant MCF-7 cell line (MCF-7_DoxR) were treated with different combination ratios of DOX, Dis, and Hyd followed by measuring the cell viability using the MTT assay. Synergism was determined using a combination index, isobologram analysis, and dose-reducing index. The anti-proliferation activity and mechanism of the triple combination were investigated by apoptosis analysis. The results showed a reduction in the IC_50_ values of DOX in MCF-7_WT cells (from 0.24 μM to 0.012 μM) and MCF-7_DoxR cells (from 1.13 μM to 0.44 μM) when treated with Dis (0.03μM), and Hyd (20μM) combination. Moreover, The triple combination DOX/Hyd/Dis induced significant apoptosis in both MCF-7_WT and MCF-7_DoxR cells compared to DOX alone. The triple combination of DOX, Dis, and Hyd showed a synergistic drugs combination to decrease the DOX dose needed to kill both MCF-7_WT and MCF-7_DoxR cancer cells and enhanced chemosensitivity to DOX.

## 1. Introduction

Breast cancer is one of the most common cancers affecting females [1]. Although the ideal therapy for each breast cancer depends on cancer stage, tumor type, and patient status [2], single-modality chemotherapies such as doxorubicin are commonly used to treat many types of breast cancer and their metastasis [3]. Doxorubicin (DOX) is an anthracycline cytotoxic drug that is highly efficient for treating cancer [4]. Unfortunately, dose escalation of DOX is detrimental due to its cardiotoxicity due to changes in the myocardium, cardiomyopathy, and heart failure that may lead to death [5]. Cancer cells also develop resistance against DOX, resulting in treatment failure [3]. However, there are different approaches to increasing the sensitivity of cells toward chemotherapy and reversing their resistance [6]. One of the most effective strategies is the combined-modality treatment composed of two or more drugs [7–9]. The underlying principle for using drugs combination is to achieve a better therapeutic effect with lower side effects. Moreover, the drug combinations target diverse mechanisms resulting in reverse drug resistance and lower side effects [10]. Two drugs combination of chemotherapies with cytotoxic activity are commonly used and are effective, especially if they show synergism [11, 12]. Many drugs affect cell epigenetic processes, an essential mechanism for maintaining normal gene expression [13]. For example, DNA methylation, histone modifications and control gene transcription and translation through microRNAs [14]. Based on that genetics and epigenetics disturbances were recognized to produce many mutations in the DNA which led to cancers, thus reversal of their damaging effects was proved to have therapeutic benefit for cancer treatment [11, 15]. Many drugs were designed to target epigenetic enzymes, and many generations were developed and classified based on their epigenetics remodelling activity [16, 17]. Accordingly, drugs that modulate epigenetic enzymes were reported to overcome chemotherapy resistance in solid tumours such vorinostat, hydralazine, valproic acid, procaine, and others [18, 19].

Many FDA approved drugs have an indication, such as hydralazine [20], were repurposed and used as adjuvants in chemotherapy regimens due to their effect in modulating epigenetic processes in cancer cells [14, 21, 22]. Hydralazine (1-Hydrazinophthalazine) is a vasodilator with smooth muscle relaxation activity and has been used to treat high blood pressure for many years [23]. Hydralazine produces lupus-like syndrome due to DNA demethylating properties. hydralazine can reduce the expression of DNA methyltransferase enzymes, DNMT1 and DNMT3a, rather than directly inhibiting their enzymatic activity [20]. It was proved that the side effects of drugs might help treat other diseases. Hypermethylation of tumor DNA leads to the silencing of suppressor gene expression; thereby, the inhibition of DNA methyl transferase enzymes will lead to the re-expression of tumor suppressor genes [24]. It was reported by Segura-Pacheco et al., that the inhibition of DNA demethylation via hydralazine reversed DOX resistance of MCF-7/Adr cells more than verapamil, a well-known P-pg inhibitor. Though, interference of DNA demethylation restores cell sensitivity to DOX [25]. Moreover, aldehyde dehydrogenase enzymes (ALDH) are over-expressed in cells that are resistant to chemotherapy. ALDH enzymes are essential for differentiation, development and stimulate resistance of cancer stem cells. Cancer stem cells have a vital role in recurrence, metastasis, and patient death. Therefore, reducing the resistant cancer stem cells will lead to a good cancer prognosis [26, 27].

Disulfiram (Tetra-ethyl-Disulfane dicarboamide) is an aldehyde dehydrogenase enzyme (ALDH) inhibitor that has been used for alcohol abuse for more than 60 years [28]. ALDH is over-expressed in cells that are resistant to chemotherapy. ALDH enzymes are essential for cancer stem cell differentiation, development, drug resistance, and relapse. Cancer stem cells are vital in recurrence, metastasis, and patient death. Therefore, reducing the resistant cancer stem cells will lead to a good cancer prognosis [26, 27]. Currently, disulfiram (Dis) is repurposed due to its anti-cancer activity especially when used with copper [29]. Disulfiram suppresses the levels of ALDH in cancer stem cells [30]. Therefore, inhibition of ALDH was supposed to reverse the resistance of these cells [31]. The anti-cancer activity of Dis is due to many mechanisms such as inhibiting cancer cells proteasome activity through the formation of a complex with copper or zinc [30]. In addition, Dis can overcome cancer cell resistance to treatment by inhibiting of P- glycoprotein (P-pg) pump, which is responsible for the efflux of drugs out of the cancer cell leading to a subtherapeutic activity of used chemotherapy [32]. Indeed, Dis has been combined with many anti-cancer agents as an adjuvant, showing the ability to enhance therapeutic potency and decrease drug resistance [33].

**Fig 1:**
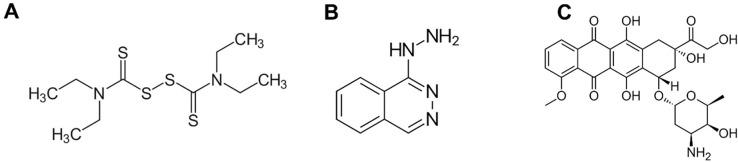
The chemical structures of disulfiram (A), hydralazine (B), and doxorubicin (C).

Based on Hyd and Dis capacity to exert anti-tumor activity and the superior efficacy of combination therapies against cancer, we combined DOX with Hyd and Dis (Fig 1) as a triple treatment in the current study to examine the synergistic effect against wild-type and DOX-resistant MCF-7 breast cancer cells, thereby, introducing a promising combined-modality approach to the treatment of breast cancer.

### 2. Materials and methods

#### 2.1. Cell culture

The parental MCF-7 (breast cancer cell line) (MCF-7_WT) was obtained from ATCC, and DOX resistance MCF-7 cell line (MCF-7_DoxR) was developed from the parental cell line at Cell Therapy Center (The University of Jordan) [34]. Cells were cultured as an attached monolayer and maintained in RPMI 1640 medium (EuroClone, Italy) supplemented with10% (v/v) heat-inactivated fetal bovine serum (FBS) (EuroClone, Italy), 1% penicillin-streptomycin (EuroClone, Italy), and 2 mM L-glutamine. Cells were incubated at 37°C in a 5% CO_2_ tissue culture incubator (Memmert, Schwabach, Germany).

### 2.2. Cell viability assay (MTT)

To determine the anti-proliferative effect of single and combinations of Doxorubicin, Disulfiram (Fisher Scientific Lab, USA), and Hydralazine (TCI, Japan) on both cell lines, IC_50_ values were measured using MTT assay. Firstly, MCF-7_WT and MCF-7_DoxR cells (9 × 10^3^ cells/well) were seeded into a 96-well plate (Corning, USA) and treated with different concentrations of DOX, Dis, and Hyd ranging from 0.001 to 100 μM. Then, cells were incubated at 37°C in a 5% CO_2_ incubator for 72 h, after which the old media was aspirated, and 10 μl of MTT salt (Bioworld, USA) in 100 μl of fresh media was added to each well. After that, plates were incubated at 37°C for 3 h, followed by adding 50 μl of solubilization solution (DMSO). The absorbance of the solution was measured at 560 nm using a Glomax plate reader (Promega, USA).

### 2.3. Combination index

To determine the effect of using combinations of DOX, Dis, and Hyd on MCF-7_ DoxR and their control cells MCF-7_WT, the combination index was calculated using Compusyn software (Version 1.0, Compusyn, Inc., and Paramus, NJ, USA). Briefly, an isobologram analysis was performed using the Compusyn software program. Cells were treated with different combinations of DOX, Dis, and Hyd either individually or in combinations of a 0.03:20.00:0.25 molar ratio, respectively, for 72 h followed by MTT assay analysis to determine the cell viability and combination index (CI). A CI of <1.0 indicates synergism, a CI of 1 indicates additive activity, and a CI > 1.0 indicates antagonism.

### 2.4. STRING database prediction of the possible direct and indirect protein interactions

A assessment of key regulatory molecules involved in inducing network interactions of the synergistic effect of Hyd, Dis, and DOX combination using the STRING database for prediction of the possible direct and indirect protein interactions [35].

### 2.5. Apoptosis by flow cytometry

To investigate the growth inhibition of all cell lines treated with disulfiram, hydralazine, and doxorubicin, the mechanism of cell death was determined by Annexin V/ Propidium iodide (PI) stain using flow cytometry. MCF-7_WT and MCF-7_DoxR cells (1 × 10^5^ cells/well) were seeded into 12-well plates and exposed to 0.5 μM DOX, 0.5 μM Dis, and 50 μM Hyd. After 24 h, the cells were trypsinized using StemPro™ Accutase™ Cell Dissociation Reagent (Gibco™, UK). Then, the collected cells were washed with PBS. After that, an Annexin V/PI apoptosis kit (R&D systems, USA) was used to stain the cell pellets following the kit’s instructions. 10,000 events were counted by BD FACS CANTO II and analyzed using BD FACS Diva™ software version 7.0.

### 2.6. Statistical analysis

Data were entered on GraphPad Prism version 7. One-way ANOVA tests were used to analyze data, and the significant difference was considered when the *p*-value < 0.05. Values in the figures represent the mean of three independent experiments ± standard deviation.

## 3. Results and discussion

When disulfiram and hydralazine were combined with DOX, their individual mechanisms of action may complement each other, leading to enhanced anti-cancer effects. For example the inhibition of ALDH by disulfiram can increase the accumulation of acetaldehyde, which in turn may enhance the production of ROS induced by hydralazine and DOX [27]. This synergistic effect of increased ROS generation and DNA damage can lead to more efficient eradication of cancer cells [36].

### 3.1. STRING modeling and simulation for prediction and understanding the dynamics and signaling at a molecular level

The mechanisms of drug synergy can be complex. Several key regulatory molecules have induced the synergistic effect of doxorubicin and disulfiram. By inhibiting poly hydroxylases (PHDs) and activating hypoxia-inducible factor (HIF), hydralazine can potentially induce a hypoxic microenvironment within the tumor, which may sensitize cancer cells to the cytotoxic effects of DOX. Hypoxia has been linked to chemoresistance, and targeting the HIF pathway might help overcome this resistance [37].

To analyze the protein interactions that are involved in increasing the chemosensitivity of cells to DOX, STRING modeling and simulation were used to understand the possible mechanism of synergism between the triple combination [35]. The interaction between multi-drug resistance and doxorubicin, a chemotherapeutic drug, can be analyzed by understanding protein interactions. This resistance often occurs due to the overexpression or altered activity of specific proteins involved in drug transport, drug metabolism, or drug-target interactions. The overexpression of drug efflux pumps, such as P-glycoprotein (P-gp), and increased detoxification by enzymes like ALDH and GSH contribute to doxorubicin resistance [34].

**Fig 2:**
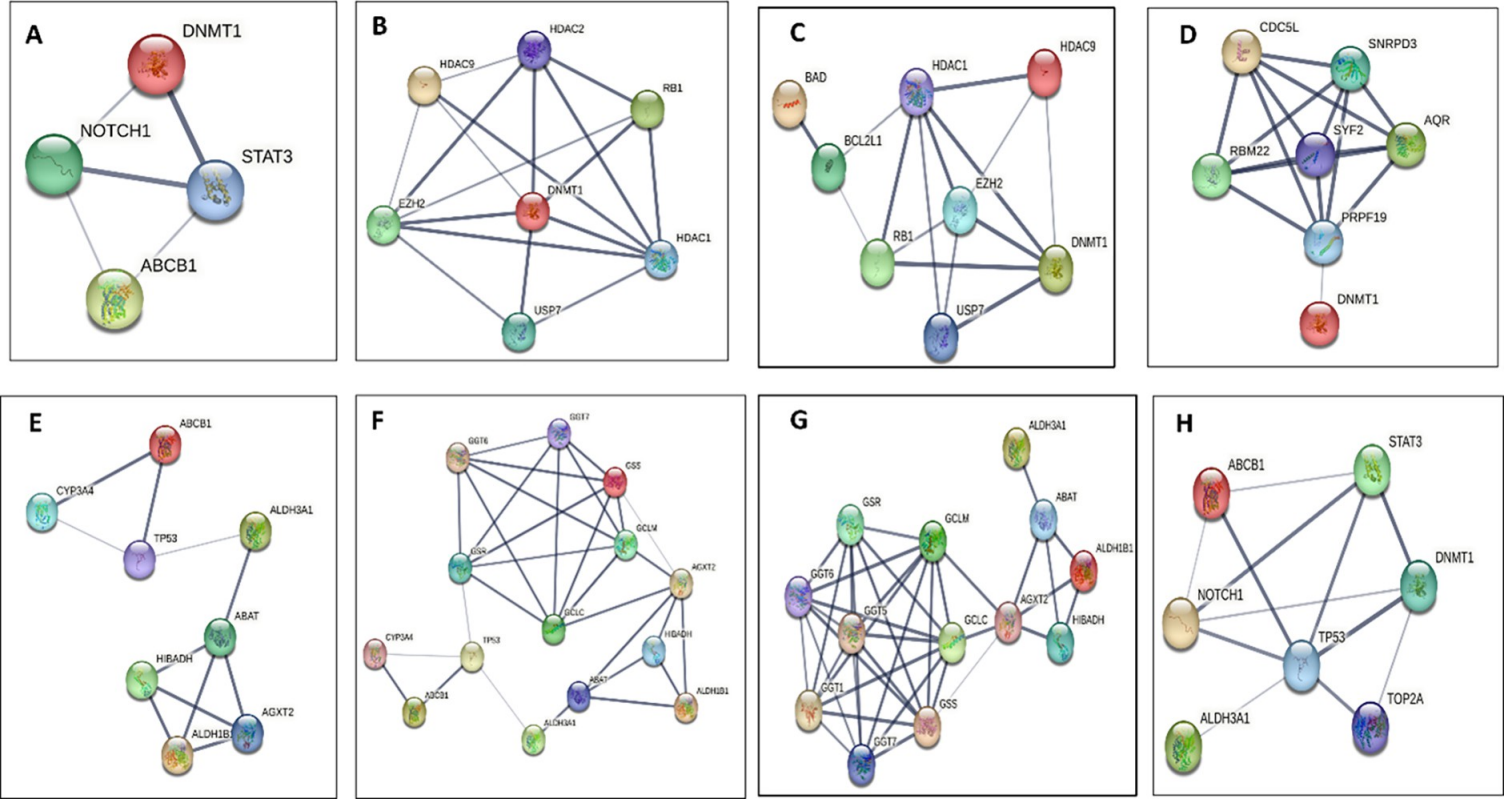
STRING analysis of protein–protein interactions. (A) Interaction between proteins involved in drug resistance STAT3, NOTCH, DNMT, (B) Illustration of combination of HDACi and DNMTi pathway, (C) combination of HDACi and DNMTi and the effect of cell apoptosis proteins, (D) interactions of DNMTi and proteins ivoved in cell cycle, (E) Effect of ALDH enzymes on P53 proteins, (F) Interactions between ALDH and glutathione (GSH), (G) Interactions between ALDH, ABCC1 and p53 proteins, (H) Signalling pathway showed interaction of DNMT, ALDH, P53, TOP2A, ABCB1. STAT3, NOCH1. (thickness of edges indicate confidence).

Walhan et. al. investigated the effect of gene silencing on the expression of STAT3, NOCH1, and ATP-binding cassette (ABC) that were involved in drug resistance, and they revealed that all of them were downregulated due to gene suppression [34]. Based on this result, inhibition of any gene that affects these proteins, such as DNMT inhibition, will decrease their expression (Fig 2A). Breast cancer resistance protein (BCRP, encoded by the ABCG2 gene) actively pumps drugs out of cancer cells, reducing their intracellular concentration and efficacy. They can also efflux doxorubicin, leading to drug resistance [8].

Hydralazine as an inhibitor of DNA methylation, can potentially enhance the DNA damage caused by DOX and impair DNA repair mechanisms. This synergistic effect may potentiate the cytotoxicity of DOX. Furthermore, combined treatment with hydralazine and DOX may enhance the suppression of Notch signaling cell sensitivity to DOX [38, 39]. Doxorubicin has been shown to inhibit HDAC activity, accumulating acetylated histones and subsequent transcriptional activation. When combined with hydralazine, which can also affect DNA methylation, the combined action on chromatin structure and gene regulation may potentiate the cytotoxic effects of doxorubicin (Fig 2B) [40]. The combined effects of doxorubicin and hydralazine on cell cycle progression may lead to enhanced cytotoxicity by preventing cancer cells from repairing DNA damage and proliferating [41] (Fig 2C).

Regarding cell cycle protein interactions, the cell cycle is a highly regulated process that involves the coordinated action of various proteins to ensure accurate cell division. Multiple regulatory steps are involved in the progression of the cell cycle. Fig 2D showed an interaction of DNMT with cell cycle proteins such as CDC5 and PRPF19 regulatory proteins [42] as well as Dis and DOX were involved in p53 signaling pathway (Fig 2G) [43].

Disulfiram, on the other hand, can inhibit DNA repair enzymes such as aldehyde dehydrogenase (ALDH), which is involved in DNA base excision repair. The combination of these drugs can impair DNA repair pathways (Fig 2E) [27], accumulating DNA damage and increasing cell death. Disulfiram reduces glutathione (GSH), making cancer cells more susceptible to the cytotoxic effects of doxorubicin [44, 45], promoting the expression of antioxidant genes and increasing the cellular defense against oxidative stress induced by DOX. Glutathione S-transferases enzymes are involved in cellular detoxification processes and can contribute to doxorubicin resistance by conjugating doxorubicin with glutathione, leading to drug inactivation and efflux (Fig 2F) [46–48]. DOX exerts its anti-cancer effects by inhibiting the activity of topoisomerase II, an enzyme involved in DNA replication and repair. However, alterations in TOP2 levels or mutations in its gene (TOP2A) can contribute to doxorubicin resistance [49] (Fig 2H).

### 3.2. The effect of disulfiram and hydralazine combination drug treatment

Breast cancer is the most diagnosed cancer and is associated with high mortality rates worldwide [50]. Doxorubicin resulted in a significant improvement in breast cancer prognosis for many years. However, the development of resistance of cancer cells to this effective drug led to decreased efficacy [51].

**Fig 3:**
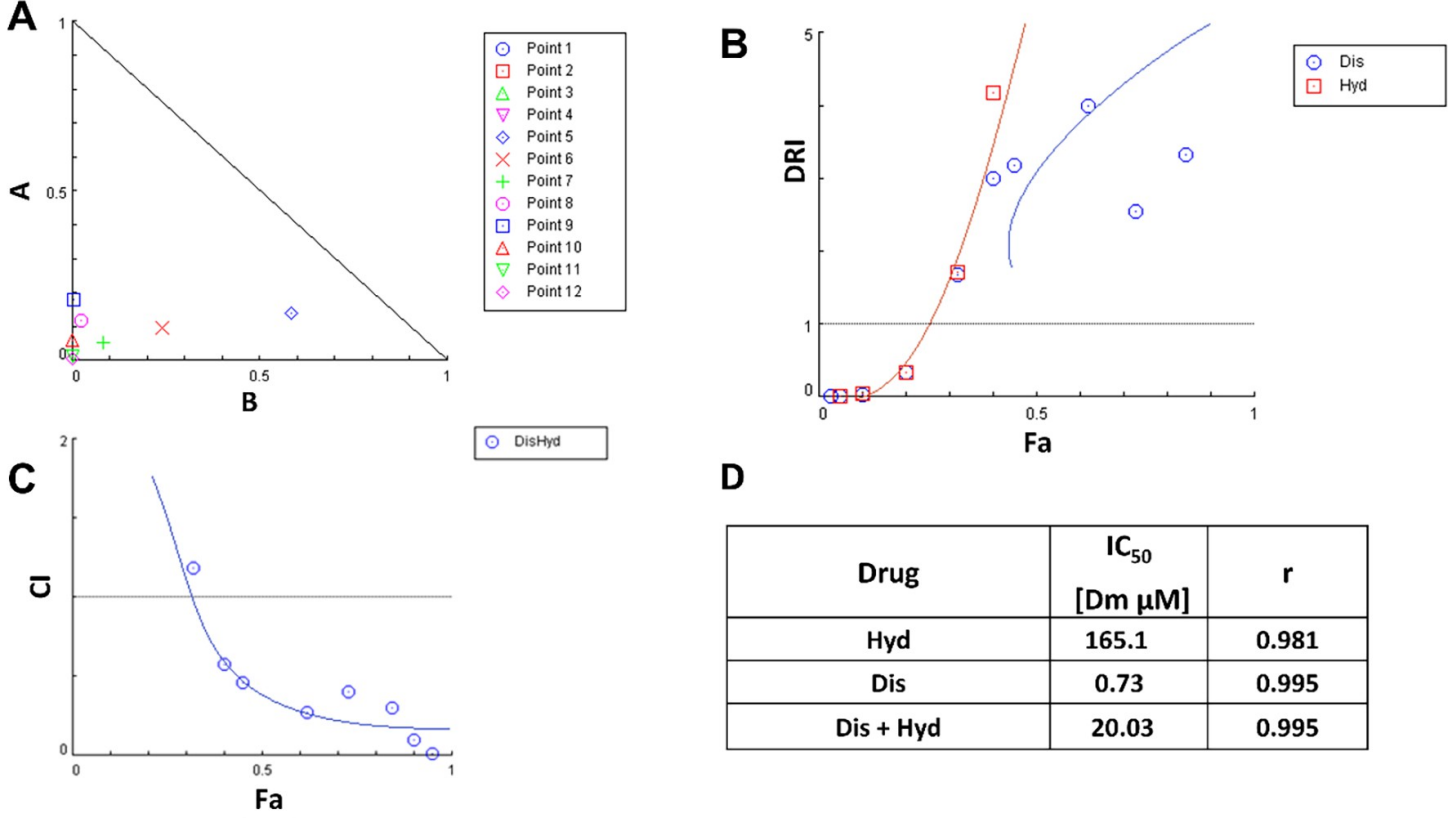
Diagnostic graphics produced for synergistic affect quantification. (A) Isobologram for Dis/Hyd combination at different concentration points, (B) The Fa-DRI plot (Chou-Martin plot) for the non-constant ratios of Dis/Hyd combination, (C) The fraction affected (Fa) versus combination index (CI) plot after treatment with Dis/Hyd combination, that most of CI values are <1 in the range 0.4–0.95, (D) Table of IC_50_ of Dis, Hyd and Dis/Hyd combination.

The effect of Dis/Hyd combination was investigated to confirm their anti-cancer activity and synergism toward wild-type MCF-7 breast cancer. Cell viability (MTT) assay was performed to explore the effect of Dis and Hyd on cell growth. MCF-7 cells were treated with increasing concentrations of both drugs separately and combined. The IC_50_ of Hyd, Dis, and the combination was found to be 165.1, 0.73, and 20.03 μM, respectively (Fig 3D). Findings from this study showed a synergistic cytotoxic effect of Dis and Hyd on MCF-7 wild type cells and a reduction of IC_50_ of Dis from 0.73 μM to 0.03 μM and Hyd from 165.1 to 20 μM (Fig 3B).

The combination of Dis and Hyd showed synergism with a combination index (CI) < 1 at Fa ranging from 0.4% to 0.95% (Fig 3C). Also, it was observed from the isobologram (Fig 3A), and drug-reducing index curve (Fig 3B) of the non-constant combination experiment of the two drugs that there is a synergism between them. The rationale behind the combination of Hyd and Dis is based on targeting various pathways, including DNA methylation, mainly by Hyd [52], and aldehyde dehydrogenase, principally by Dis, to reduce DOX resistance [53]. The combination of Dis and Hyd was investigated to exclude any interference between the two drugs or antagonism before evaluating their combination with DOX against DOX-resistant cells. The combination of Dis and Hyd showed good synergism and acceptable IC_50_ values.

### 3.3. Effect of Disulfiram and Hydralazine combination on induction of cell apoptosis

**Fig 4:**
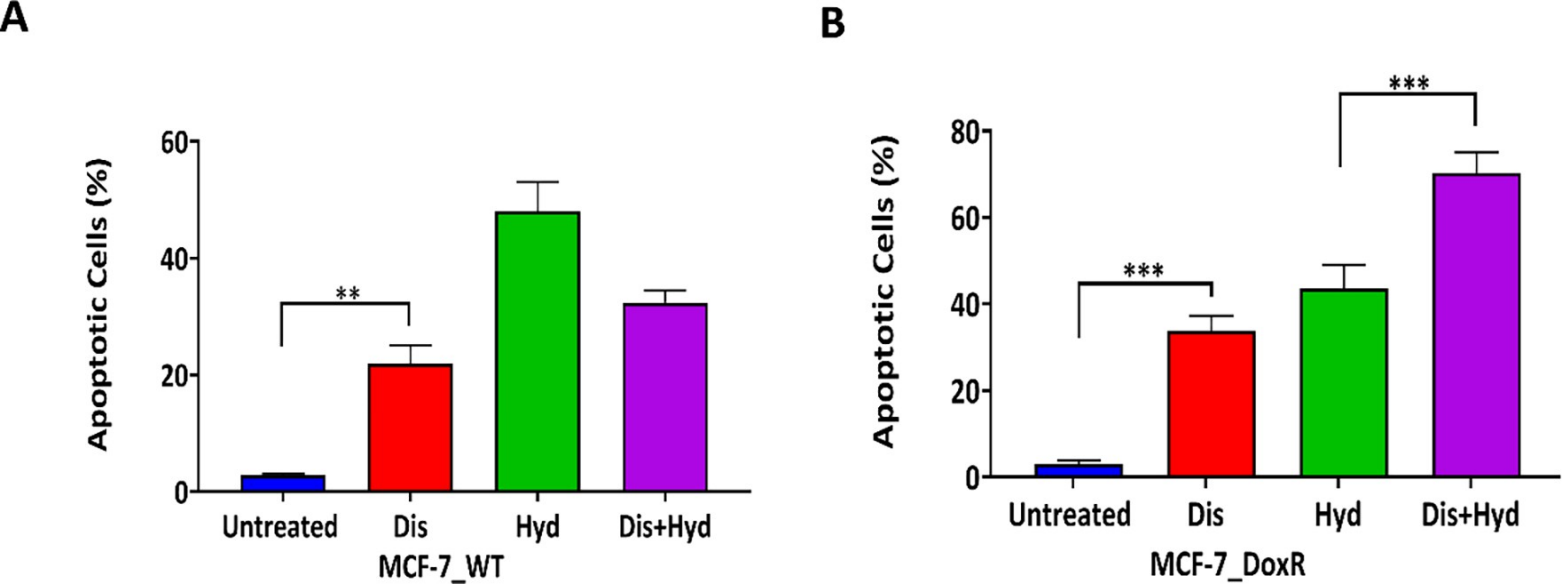
A) Induced apoptosis of MCF-7_WT cells treated with Dis and Hyd alone and combined B) Induced apoptosis of MCF-7_DoxR cells treated with Dis and Hyd alone and combined.

The mechanism of cell death was determined by investigating the effect of Dis/Hyd combination on apoptosis. Combining the two drugs enhanced apoptosis compared to either Hyd or Dis on MCF-7_DoxR cells but not on MCF-7_WT cells (Fig 4). There was a statistically significant difference between the apoptotic cell percentage of Dis/Hyd combination in MCF-7_DoxR cells (70.3% vs 32.4%) with p values < 0.001, (Fig 4). A possible explanation for this increase in apoptosis might be due to the apoptosis crucial role in resistance [54], and drugs that target the process will have a profound effect on MCF-7_DoxR cells (Fig 4B), compared to MCF-7_WT cells (Fig 4A). The effect of Hyd on apoptosis was higher than that of Dis, in both MCF-7_WT and MCF-7_DoxR cells. Ruiz-Magaña et al showed that Hyd induced caspase-dependent apoptotic cell death in leukemic T cells and the dependence of this process on the mitochondrial death pathway [43]. Similarly, drug-resistant MCF-7 breast cancer cells induced by doxorubicin show hypo- and hypermethylation processes, which augment each other in many pathways [52]. Additionally, chemotherapeutic drug-induced DNA-hypermethylation affects the tumor cells respond to the cytotoxicity mediated by cancer stem cell-like transition more than ALDH inhibition [55].

### 3.4. Effect of DOX/ Dis/ Hyd triple combination on resistant MCF-7 cancer cell line

Following the successful confirmation of synergism between Dis/Hyd combinations, the effect of the triple drug combination (DOX/Dis/Hyd) on cell growth inhibition was explored on MCF-7_WT and MCF-7_DoxR cells.

Combining drugs to treat cancer is an effective strategy to overcome drug resistance, especially if the combined drugs are relatively safe and show synergistic effects [56]. This study evaluated a triple combination of Dis/Hyd and the commonly used anti-cancer doxorubicin. Synergism or antagonism was governed using the combination index method, isobologram, and curve-shift analysis. Fraction-affected (Fa) values were determined after treatment with different concentrations of drug combinations, then the combination index (CI) and drug reduction index (DRI) were calculated for each Fa value.

**Fig 5:**
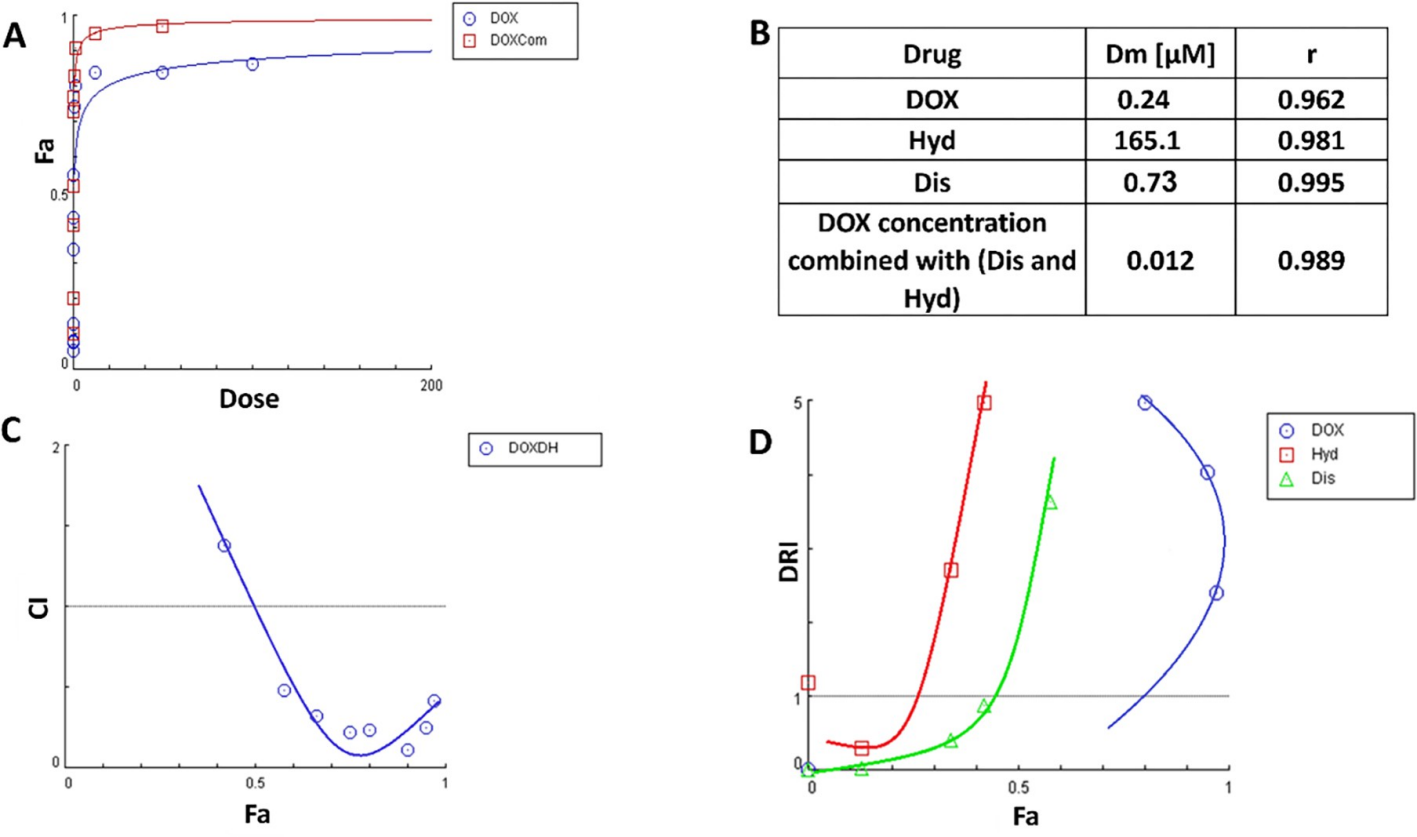
Diagnostic graphics produced for synergistic effect quantification of DOX/Dis/Hyd combination against MCF-7_WT cells. (A) Dose -Fa curve for DOX alone and for DOX with (DOX/Dis/Hyd combination) at different concentration points (B) Table of IC_50_ of DOX, Dis, Hyd alone and combination (C) The fraction affected (Fa) versus combination index (CI) plot after treatment with DOX/Dis/Hyd combination, that most of CI values are <1 for the range of 0.5–0.95(D) The Fa-DRI plot for the non-constant ratios of DOX/Dis/Hyd combination.

**Fig 6:**
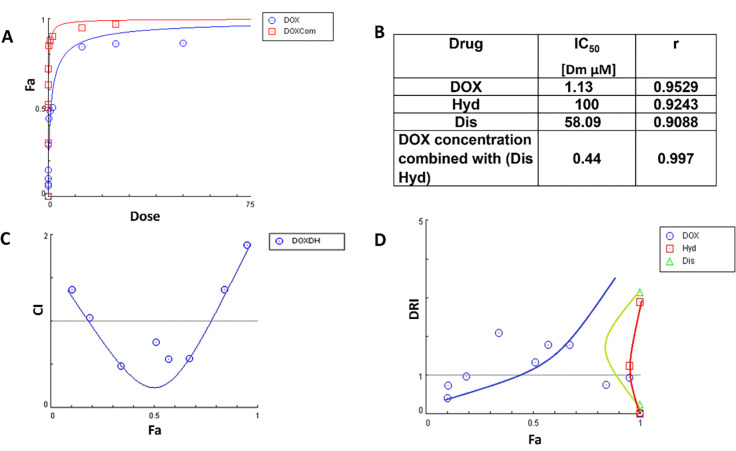
Diagnostic graphics produced for synergistic effect quantification of DOX/Dis/Hyd combination against MCF-7_DoxR cells. (A) Dose -Fa curve for DOX alone and for DOX with (DOX/Dis/Hyd combination) at different concentration points (B) Table of IC50 of DOX, Dis, Hyd alone and combination (C) The fraction affected (Fa) versus combination index (CI) plot after treatment with DOX/Dis/Hyd combination, that most of CI values are < 1 for the range of 0.25–0.8 (D) The Fa-DRI plot for the non-constant ratios of DOX/Dis/Hyd combination.

MCF-7_WT and MCF-7_DoxR cells were treated with a combination of (Dis 0.03 μM and Hyd 20 μM) and different concentrations of doxorubicin, and the results of the three drugs are shown in Figs 5 and 6. The results showed good synergism between the three drugs (CI<1). It was found that IC_50_ the concentration of DOX decreased from 0.24 to 0.012 μM in MCF-7_WT cells (Fig 5).

The three combined drugs were investigated on (180 nM) MCF-7_DoxR cell line. The cytotoxic effect of DOX combined with Dis (0.03μM) and Hyd (20μM) was found to be higher than DOX alone in resistance cells (Fig 6). The IC_50_ of DOX in the resistance cells decreased from 1.13 μM to 0.44 μM (Fig 6). The differences in the decrease in IC_50_ of wild type compared to resistant cells (20-fold vs 2.7- fold) can be explained by the presence of other important signalling pathways that are pivotal in resistant cells due to their unique characteristics.

Any breakthrough that enhances the sensitivity of resistant cells to chemotherapy has the potential to significantly impact cancer treatment and patient outcomes. Understanding the physiological relevance of increasing sensitivity to chemotherapy in resistant cells could potentially contribute to the development of personalized treatment strategies [57, 58]. By identifying specific molecular targets or pathways associated with resistance and sensitivity, clinicians may be able to tailor treatment approaches to individual patients, leading to more effective and precise therapies [57]. Resistance to chemotherapy is a complex process with multiple mechanisms and is a major cause of chemotherapy failure and disease recurrence [51, 59]. New strategies are continuously explored to overcome resistance and improve the sensitivity to chemotherapy, including repurposing known medications as anti-cancer alone or combined with chemotherapeutic agents [60, 61]. Many studies were performed to evaluate the combination of different medications with DOX against breast cancer. For example, a study conducted by Tun et al., investigated the therapeutic synergism between renieramycin M and doxorubicin in MCF-7 breast cancer cells. They revealed that the value of DOX (IC_95_) reduced eight-folds after combination with renieramycin [62].

**Fig 7:**
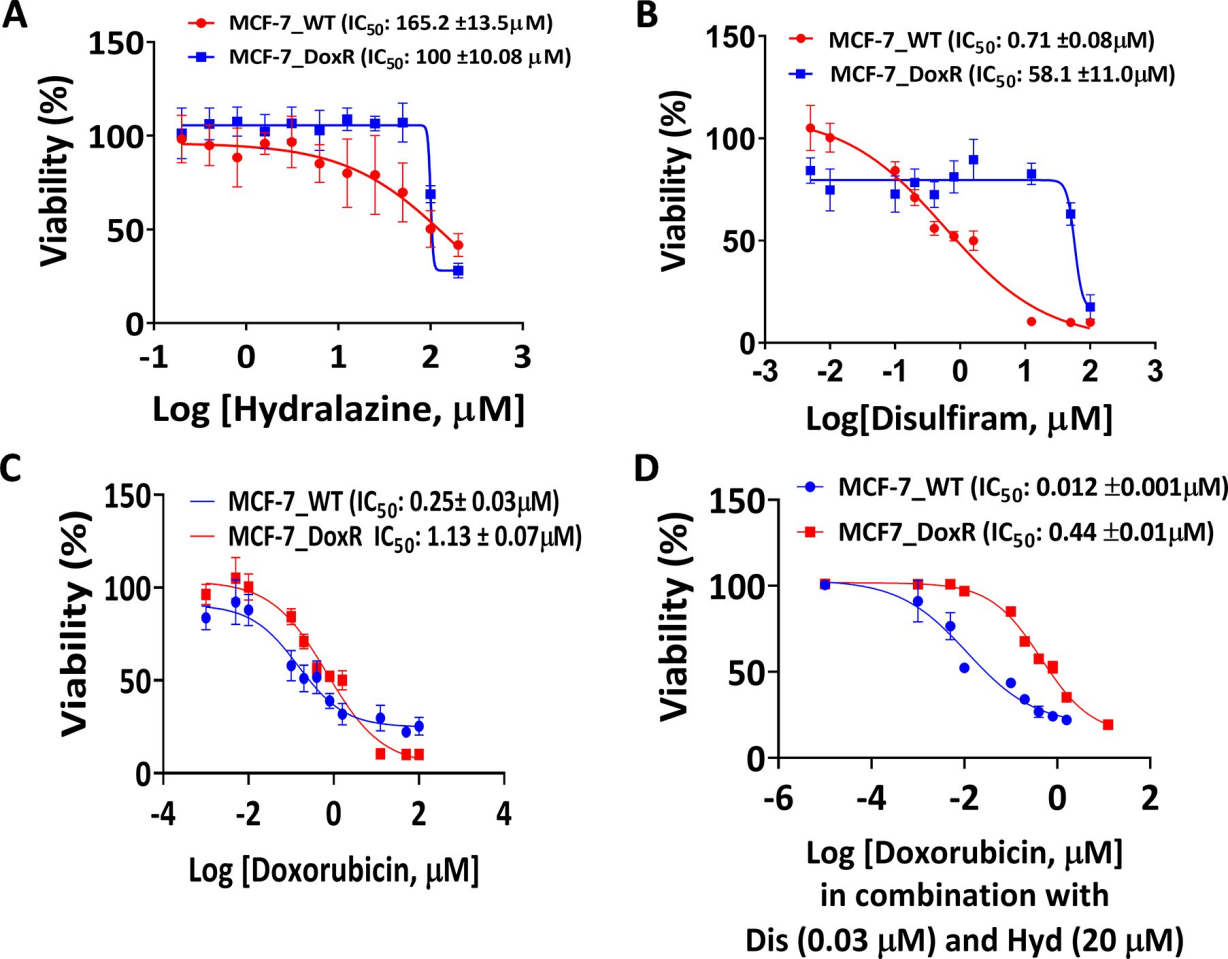
IC_50_ values after treatment with DOX/Dis/Hid. The MCF-7_WT and MCF-7_DoxR cells were treated with DOX, Dis, Hyd and DOX/Dis/Hyd to assess the cytotoxicity levels (A) The dose-response curve for cells treated with Hyd and; (B) The dose-response curve for cells treated with Dis; (C)The dose-response curve for cells treated with DOX; (D) The dose-response curve for cells treated with DOX/Dis/Hyd in combination, the curve showing DOX IC_50_ with and without combination with Dis/Hyd (0.03/20 μ*M)*. All cytotoxicity values represent the average ± SD of three independent experiments.

It was reported that Dis synergizes DOX effect on cell growth of triple-negative breast cancer [63], hepatocellular carcinoma cells [64], and on DOX resistant cells through liposomes co-encapsulation of Dis and DOX [33]. Hyd, in combination with valproate, was extensively studied in clinical studies to reduce the resistance of DOX and other chemotherapies [64–66]. However, to our knowledge, the effect of the combination of DOX/Dis/Hyd on DOX resistance has not been examined. Fig 7 demonstrates that DOX/Dis/Hyd combination resulted in a higher impact on growth inhibition for both MCF-7_WT and MCF-7_DoxR cells.

### 3.5. Effect of DOX/ Dis/Hyd/triple combination on induction of cell apoptosis

**Fig 8:**
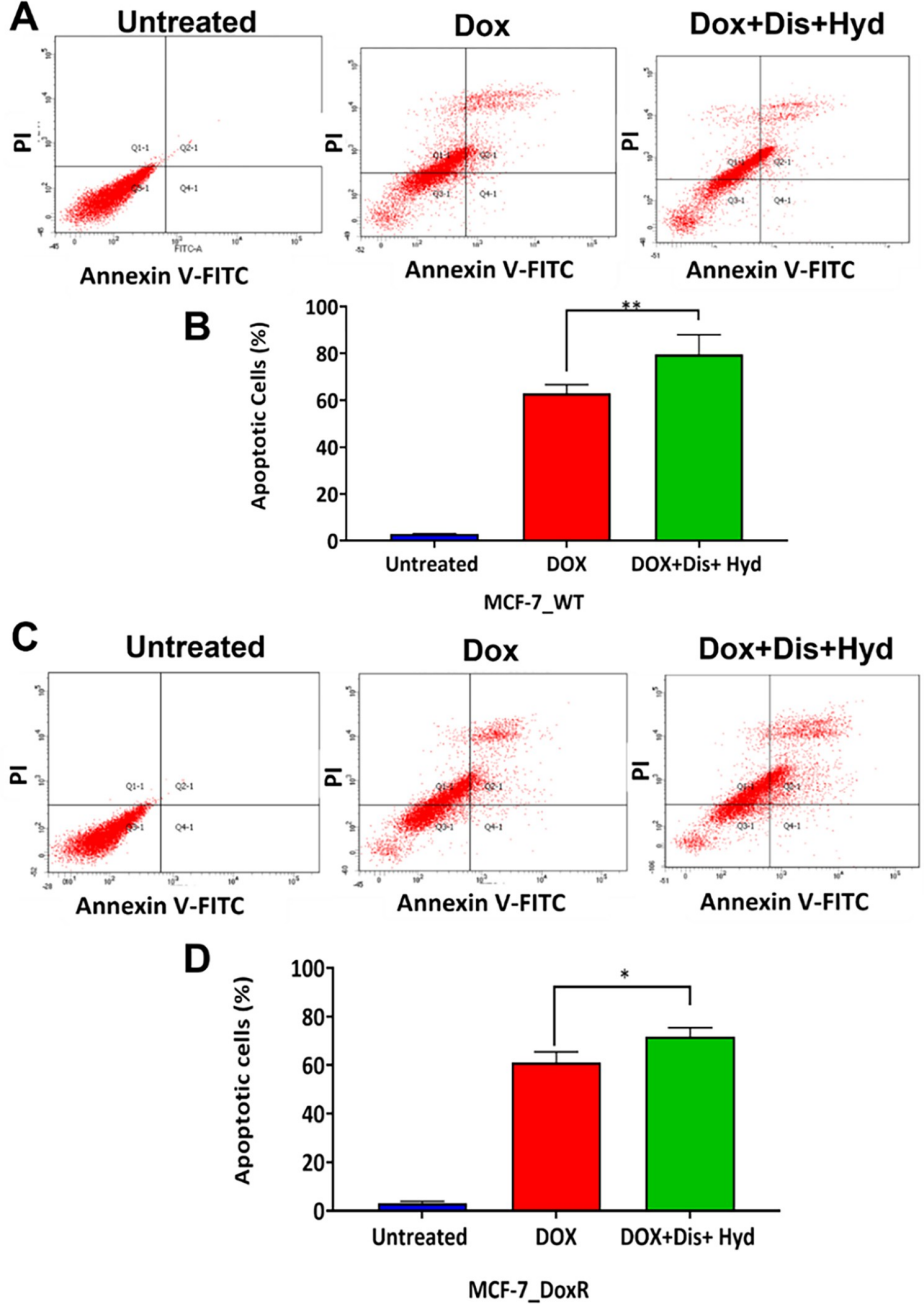
Apoptosis Induced by DOX/Dis/Hyd single treatment and combinations. A) and B) MCF-7_WT cells, C) and D) MCF-7_DoxR cells.

The effect of DOX/Hyd/Dis combination on the mechanism of cell death was determined using an apoptosis assay. The triplet combination DOX/Hyd/Dis induced statistically significant apoptosis in both wild type and resistance compared to DOX alone, 79.5% compared to 63.05% in the wild type and 71.6% compared to 61.35% in the resistant cells, p-value = 0.002 and 0.03, respectively (Fig 8).

## 4. Conclusion

In conclusion, this study showed for the first time a synergetic effect of DOX/Dis/Hyd combination against MCF-7 breast cancer cells proliferation *in vitro*. This triple combination might offer new methods of improving treatment therapy of doxorubicin in resistant tumor cells and reduce the dose of the chemotherapeutic drug. Future work will involve combinations of these two promising anti-cancer drugs with other drugs with well-known safety profiles to provide new strategies to combat chemotherapy resistance. Using a Xenograft experiment of human breast cancer will provide more clarity regarding the therapeutic efficacy of the triple (DOX/Dis/Hyd) combination. It’s important to note that the use of disulfiram and hydralazine in cancer treatment is still under investigation, and their combined mechanism of action may vary depending on the specific cancer type and context. Clinical trials and further research are necessary to fully understand and optimize the potential synergistic effects of this drug combination. Clinical trials and further research are necessary to fully understand and optimize the potential synergistic effects of this drug combination.
